# Extracellular LGALS3BP: a potential disease marker and actionable target for antibody–drug conjugate therapy in glioblastoma

**DOI:** 10.1002/1878-0261.13453

**Published:** 2023-06-07

**Authors:** Beatrice Dufrusine, Emily Capone, Sara Ponziani, Rossano Lattanzio, Paola Lanuti, Francesco Giansanti, Vincenzo De Laurenzi, Stefano Iacobelli, Rodolfo Ippoliti, Annunziato Mangiola, Gianluca Trevisi, Gianluca Sala

**Affiliations:** ^1^ Department of Innovative Technologies in Medicine & Dentistry University “G. D'Annunzio” of Chieti‐Pescara Italy; ^2^ Center for Advanced Studies and Technology (CAST) Chieti Italy; ^3^ Department of Bioscience and Technology for Food Agriculture and Environment University of Teramo Italy; ^4^ Department of Life, Health and Environmental Sciences University of L'Aquila Coppito Italy; ^5^ Department of Medicine and Aging Sciences University “G. D'Annunzio” of Chieti‐Pescara Chieti Italy; ^6^ MediaPharma s.r.l. Chieti Italy; ^7^ Department of Neurosciences, Imaging and Clinical Sciences “G. D'Annunzio” University Chieti Italy; ^8^ Neurosurgical Unit Santo Spirito Hospital Pescara Italy

**Keywords:** antibody–drug conjugates, extracellular vesicles, glioblastoma, LGALS3BP, liquid biopsy

## Abstract

Glioblastoma multiforme (GBM) is a lethal disease characterized by an overall survival of about 1 year, making it one of the most aggressive tumours, with very limited therapeutic possibilities. Specific biomarkers for early diagnosis as well as innovative therapeutic strategies are urgently needed to improve the management of this deadly disease. In this work, we demonstrated that vesicular galectin‐3‐binding protein (LGALS3BP), a glycosylated protein overexpressed in a variety of human malignancies, is a potential GBM disease marker and can be efficiently targeted by a specific antibody–drug conjugate (ADC). Immunohistochemical analysis on patient tissues showed that *LGALS3BP* is highly expressed in GBM and, compared with healthy donors, the amount of vesicular but not total circulating protein is increased. Moreover, analysis of plasma‐derived extracellular vesicles from mice harbouring human GBM revealed that LGALS3BP can be used for liquid biopsy as a marker of disease. Finally, an ADC targeting LGALS3BP, named 1959‐sss/DM4, specifically accumulates in tumour tissue, producing a potent and dose‐dependent antitumor activity. In conclusion, our work provides evidence that vesicular LGALS3BP is a potential novel GBM diagnostic biomarker and therapeutic target deserving further preclinical and clinical validation.

## Graphical abstract

*LGALS3BP* is overexpressed in glioblastoma multiforme (GBM) and is highly enriched in GBM‐derived extracellular vesicles. We established a panel of patient‐derived GBM cell lines and developed a cell‐derived xenograft preclinical model. Our data reported that extracellular LGALS3BP can be used as a potential liquid biopsy GBM marker and can be efficiently targeted by a specific antibody–drug conjugate.

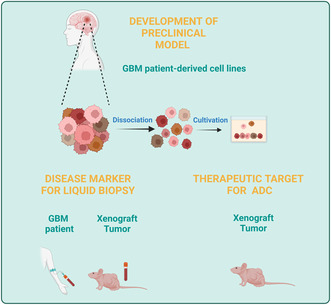

**Abbreviations**
ADC: antibody–drug conjugate
BBB: blood–brain barrier
DAR: drug–antibody ratio
DMEM: Dulbecco's modified eagle medium
EVs: extracellular vesicles
GBM: glioblastoma multiforme
IHC: immunohistochemical
KIF: kifunensine
LGALS3BP: galectin‐3‐binding protein
MRI: magnetic resonance imaging
MTT: 3‐(4,5‐dimethylthiazole‐2‐yl)‐2,5 diphenyltetrazolium bromide
OS: overall survival
ROC: receiver operating characteristic
TME: tumour microenvironment
TMZ: temozolomide
TUN: tunicamycin

## 1 Introduction

Glioblastoma multiforme (GBM) is one of the most aggressive types of cancer that initiates in the brain. Despite advancements in our understanding of GBM pathogenesis, the development of new diagnostic tools and innovative targeted therapeutics, GBM remains an incurable disease with a median overall survival (OS) approximately ranging from 7 to 15 months [1, 2]. The lack of an effective treatment has been linked to different factors, including target selection, tumour heterogeneity, immunosuppressive tumour microenvironment (TME) and poor penetration of therapeutic agents through the blood–brain barrier (BBB) [2, 3, 4, 5]. Maximal safe resection followed by adjuvant chemotherapy has remained the standard treatment for GBM [6, 7, 8]. Nonetheless, local recurrence is an inevitable event in the natural history of GBM with most patients experiencing it 6–9 months after primary treatment [9, 10]. Recurrent GBM poses great challenge to manage with no effective and well‐defined management protocols. Due to the absence of effective surgical and medical treatments currently available for advanced GBM, the identification of early diagnostic and prognostic biomarkers appears of key importance to improve the survival rate of patients and to develop new personalized treatments. Indeed, the majority of GBM patients are diagnosed when the tumour is advanced, therefore making surgery and therapy barely effective [2, 11]. Early diagnosis of GBM is challenging mainly because this malignancy most often gives non‐specific symptoms which are in common with benign brain lesions [12]. In the recent past, efforts have been made for the identification of serum/circulating biomarker suitable for liquid biopsy to be used for cancer early diagnosis and therapy response [13, 14]. Among tumour biomarkers, those deriving from TME are of particular relevance [15, 16]. As an example, it has been proposed that vesicular PD‐L1, an important immune checkpoint which can be targeted by immunotherapy, may be used as biomarker for anti‐PD‐1 therapy response in melanoma [17, 18]. It is now a well‐defined paradigm that cancer cells produce and secrete proteins able to activate the TME towards a permissive condition for growth and invasion [15, 16, 19]. Importantly, some of secreted proteins are released by cancer cells through extracellular vesicles (EVs) compartments [20]. In this respect, secreted proteins which are found to be enriched in cancer‐associated EVs have been shown to have a prime role in initiating tumour–stroma interaction [21, 22], particularly in the context of GBM progression [23, 24, 25]. Indeed, the communication between glioma and stromal cells can be induced by EVs, which, in turn, promote tumour progression through activation of fundamental processes such as active proliferation, neo‐angiogenesis, changes in cellular metabolic activity, immune escape and tumour microenvironment organization [24, 25]. Moreover, EVs freely can cross the intact BBB in both directions, thus GBM‐derived EVs can be detected in peripheral blood [26, 27]. It is, therefore, remarkable that the presence of heterogeneous membrane‐bound EVs released by cancer cells in the bloodstream may represent an innovative and promising tool for liquid biopsy, especially for brain tumours. LGALS3BP is a glycosylated protein expressed and secreted by cancer cells which have emerged as a key player in regulating cancer–stroma interaction, being one of the most abundant surface components of cancer‐derived EVs [28]. A study by Rana et al. [29] reported that plasma vesicular LGALS3BP levels correlate with glioma tumour grade, making this protein a potential biomarker for early detection. Additionally, our recent works documented that LGALS3BP targeting antibody–drug conjugate (ADC) showed potent therapeutic activity against melanoma and neuroblastoma xenografts [30, 31].

Here, we report the results of experiments aiming at demonstrating the dual role of LGALS3BP in GBM: (a) as a biomarker for liquid biopsy and (b) as a therapeutic target for an ADC. We investigated the expression of plasma vesicular LGALS3BP in a cohort of patients and healthy donors. Furthermore, we established a panel of patient‐derived GBM cell lines and developed a cell‐derived xenograft preclinical model to validate EVs‐associated LGALS3BP as potential disease marker and therapeutic target.

## 2 Materials and methods

### 2.1 Reagents

Antibodies used in the present study for immunohistochemistry and western blotting were as follows: anti‐CD63 (CBL553; Millipore, Darmstadt, Germany), anti‐β‐actin (Sigma Aldrich Corporation, St. Louis, MO, USA), anti‐CD9 (sc‐59140; Santa Cruz Biotechnology), anti‐Alix (MA1‐83977; Invitrogen, Life Technologies, Carlsbad, CA, USA) and anti‐LGALS3BP (AF2226; R&D Systems, Minneapolis, MN, USA). Dulbecco's Modified Eagle Medium (DMEM), FBS and penicillin–streptomycin (pen‐strep) were purchased from Gibco, Life Technologies, Toulouse, France and Primocin from InvivoGen. Collagenase, hyaluronidase, 3‐(4,5‐Dimethylthiazole‐2‐yl)‐2,5 diphenyltetrazolium bromide (MTT), kifunensine (KIF) (#K1140), Tunicamycin (TUN) (#T7765) and OSMI‐1 (#SML1621) were purchased from Sigma‐Aldrich Corporation.

### 2.2 Patients and specimen collection

In accordance with the Declaration of Helsinki and the protocols approved by the ethical review board, 20 patients with suspected high‐grade glioma at preoperative Magnetic Resonance Imaging (MRI) undergoing open microsurgical resection at Neurosurgical Unit of ‘Santo Spirito’ Hospital in Pescara between May 2020 and May 2021, were enrolled for plasma and fresh tissue sampling. In all cases, histopathological confirmation of GBM diagnosis was obtained. Plasma samples were collected preoperatively, soon before accessing the surgical room. Fresh tumour specimens were collected under surgical microscope view with the assistance of neuronavigation and 5‐aminolevulinic acid fluorescence to ensure a correct targeting of metabolically active tumoural area showing contrast enhancement at preoperative MRI.

To increase the sample of histopathological evaluation of LGALS3BP expression in GBM, we retrospectively retrieved 36 more paraffin‐fixed glioblastoma cases who underwent surgical resection in the previous 2 years. Clinical and radiological data were collected for all patients.

The study was approved by Chieti‐Pescara Local Ethics Committee (E.C. number 08/21.05.2020). All patients gave written consent to the study.

### 2.3 GBM patient‐derived cell lines characterization

Surgically removed tumour specimens were placed in growth medium, DMEM, containing 10% FBS and 1% pen‐strep prior to transfer on ice. Fresh samples were washed with PBS containing 1% pen‐strep, centrifuged at 500 ***g*** for 5 min, minced with scalpel and incubated in serum‐free DMEM with a mixture of collagenase and hyaluronidase for enzymatic dissociation at 37 °C for a time depending on the size of tumour specimen. Enzymatic digestion was stopped with complete medium, followed by centrifugation at 1500 r.p.m. for 10 min. The dissociated cells were washed with PBS, centrifuged at 1500 r.p.m. for 10 min and filtered with 0.45 μm cell strainer in order to discard any undigested tissues. Isolated cells were resuspended in DMEM containing 10% FBS, 1% pen‐strep and 0.25 mg·mL^−1^ Primocin and placed on vessel for culture. Cell lines were named with Gch acronym and progression number. Out of 20 GBM tissues, we successfully obtained 12 GBM patient‐derived primary cell lines (~60%). The remaining tumour samples did not attach after disaggregation or resulted in short‐term primary cell cultures, not successfully storable. After cell lines were established, cells exhibited characteristics of two‐dimensional cell line cultures, where cells flattened in a monolayer on the bottom of the culture vessel. A piece of each sample was stored at −80 °C for later acid nucleic and protein extraction and another piece was fixed with formalin and embedded in paraffin for histological analysis.

For the growth curves, cells at the same passage were seeded in six‐well plates at 5 × 10^4^ cells/well. Cells were digested with trypsin, stained using 0.4% Trypan blue and counted in haemocytometer each day for 4 days. Three independent experiments were performed in triplicate. The growth curves of the cells were plotted.

### 2.4 Cytotoxicity assays

Cell proliferation was assessed by MTT [3‐(4,5‐dimethyldiazol‐2‐yl)‐2,5‐diphenyl tetrazolium bromide] assay. Gch6 and Gch14 patient‐derived cells were seeded in 24‐well plates at a density of 1 × 10^4^ cells/well under standard growth conditions for 24 h at 37 °C in 5% CO_2_. Afterwards, cells were treated with free SH‐DM3 or SH‐DM4 at indicated concentrations for 5 days. At the end of the incubation period, cells were incubated with 250 μL of MTT solution (serum‐free medium with 0.5 mg·mL^−1^ of MTT) for further 2 h. After the removal of MTT solution, 200 μL of dimethyl sulfoxide was added to the wells for 10 min and the absorbance at 570 nm was measured using a multi‐plate reader.

### 2.5 Cellular treatments with glycosylation inhibitors

Gch6 patient‐derived cells were seeded in 60‐mm dishes at the density of 1 × 10^6^/dish under standard growth conditions for 24 h at 37 °C in 5% CO_2_. Afterwards, cells were treated with KIF (5 μm) or TUN (10 μg·mL^−1^) or OSMI‐1 (50 μm) for 48 h in serum‐free medium. At the end of the incubation period, the supernatants were collected, centrifuged at 3000 ***g*** for 10 min at 4 °C and stored at −80 °C until further usage and cells were collected and analysed by western blotting.

### 2.6 Quantitative real‐time reverse transcription–polymerase chain reaction analysis

The RNA from GBM patient‐derived cells was isolated using RNeasy® Mini Kit (QIAGEN, Courtaboeuf Cedex, France), followed by cDNA synthesis using RT2 First Strand Kit (QIAGEN) according to the manufacturers' protocols. The obtained cDNA was taken for real‐time PCR using SsoAdvanced Universal SYBR Green Supermix (Bio‐Rad, Berkeley, CA, USA) on CFX Real‐Time PCR Detection Systems. Primers were designed using primer 3 software (http://bioinfo.ebc.ee/mprimer3/) and were as follows: LGALS3BP (NM_005567.4) Fw 5′‐GAACCCAAGGCGTGAACGAT‐3′; Rev 5′‐GTCCCACAGGTTGTCACACA‐3′, and human β‐actin Fw 5′‐CAGCTCACCATGGATGATGATATC‐3′ and Rev 5′‐AAGCCGGCCTTGCACAT‐3′ as housekeeping gene. The following PCR program was used: 95 °C for 30 s and 40 cycles of 95 °C for 15 s, 60 °C for 30 s and 72 °C for 30 s. All gene expression were normalized using human β‐actin as housekeeping gene. Differences in threshold cycle (*C*_t_) number were used to quantify the relative amount of PCR target genes. Relative amounts of different gene transcripts were calculated by the ΔΔ*C*_t_ method and were converted to relative transcription ratio ($2^{-\Delta \Delta C_{t}}$) for statistical analysis.

### 2.7 Confocal microscopy

Glioblastoma multiforme patient‐derived cultured under standard growth conditions were plated at 70% of confluence on glass coverslips and after 24 h were incubated with 10 μg·mL^−1^ of anti‐LGALS3BP (1959) at 37 °C for 90 min in PBS/BSA 3%. Afterwards, the cells were washed in PBS, fixed in 4% paraformaldehyde and incubated with goat serum 5% in PBS as a blocking solution for 60 min at RT. Finally, cells were stained with AlexaFluor‐488 conjugated anti‐human IgG 1 : 100 (A11013; Invitrogen, Life Technologies) and mounted using ProLong Gold Antifade Mountant with DAPI (P36935; Thermo Fisher Inc., Waltham, MA, USA). Confocal images were acquired using a Zeiss LSM800 inverted confocal microscope system (Carl Zeiss, Gottingen, Germany); detector gain voltages and pinhole were set at the beginning of the experiment and maintained constant during the acquisition of all samples.

### 2.8 Generation, purification and DAR calculation of 1959‐sss/DM3 and 1959‐sss/DM4

1959‐sss/DM3 and 1959‐sss/DM4 ADCs were obtained as described [30]. All ADC batches after purification were analysed by SDS/PAGE and SEC. For drug–antibody ratio (DAR) determination, HIC was performed as previously described [30].

### 2.9 *In vivo* studies

For *in vivo* study, female athymic CD‐1 nu/nu mice were ordered by Charles River Laboratories (Calco, Italy) and arrived at the age of 5–7 weeks. Mice were kept under specific pathogen‐free conditions. Room temperature was at 22 ± 2 °C and relative humidity 50 ± 15%. Cages, bedding and food were autoclaved before use. Mice were provided with a standard diet and water *ad libitum* and acclimatized for 2 weeks before the start of the experiments. Housing and all procedures involving the mice were performed according to the protocol approved by the Institutional Animal Care and Use Committee of the Italian Ministry of Health (Authorization no. 1118/2020). Xenografts were generated by injecting subcutaneously into the right flank of mice a suspension of 1 × 10^6^ Gch6 cells in a total volume of 200 μL of PBS and Matrigel (ratio 1 : 1). For therapeutic studies when xenografts became palpable (approximately 100 mm^3^), animals were divided into groups to provide a similar range of tumour sizes for each group. The treated group received intravenous injections of 1959‐sss/DM3 or 1959‐sss/DM4 twice weekly for a total of three injections at the indicated doses, whereas the control group received PBS only. The mice weight and the tumour volume were monitored weekly by a calliper and calculated using the following formula: tumour volume (mm^3^) = (length × width^2^)/2. A tumour volume of 1 cm^3^ was chosen as an endpoint for all experiments after which mice were sacrificed and tumours dissected, fixed with formalin and embedded in paraffin. For biodistribution studies, when xenografts became palpable (approximately 100 mm^3^), animals were divided into groups to provide a similar range of tumour sizes for each group. The treated group received one single intravenous injection of 1959‐sss/DM4 at the dose of 10 mg·kg^−1^, whereas the control group received PBS only. Mice were sacrificed at 24, 48 and 72 h, and tumour, lungs, liver, kidney and spleen were collected for biodistribution analysis by ELISA. Finally, for EVs correlation studies in mice, when xenografts became approximately 300, 600 and 1200 mm^3^, mice were sacrificed and blood was collected for EVs isolation.

### 2.10 Serum‐ and cell‐derived extracellular vesicles purification

As concerns cell‐derived EVs isolation by ultracentrifugation, confluent GCh6 and GCh14 cells were cultivated for 48 h in serum‐free medium. The glycosylation inhibitor KIF was added to confluent Gch6 cells at 5 μm for 48 h in serum‐free medium. Around 100 mL of supernatant was collected and differential ultracentrifugation was performed for EVs isolation [32]. Briefly, supernatant was centrifuged at 300, 2000, 10 000 and 100 000 ***g*** for 10, 30 and 70 min, respectively, at 4 °C. The pellet of the last centrifugation consisted of crude EVs, which were analysed by western blotting and ELISA for LGALS3BP and classical EVs markers expression. For human and mice serum‐derived EVs isolation, ExoQuick Exosome Precipitation Solution (Cat# EXOQ20A‐1; System Biosciences, Palo Alto, CA, USA) was used following the manufacturer's protocol [33, 34, 35, 36]. Briefly, blood samples were centrifuged at 3000 r.p.m. for 15 min at 4 °C, then 63 μL of ExoQuick Solution was added to the serum (1 : 4) and incubated for 30 min at 4 °C. Suspensions were centrifuged at 1500 ***g*** for 30 min and EVs pellets were resuspended in 100 μL of PBS and analysed by western blotting and ELISA for LGALS3BP and classical EVs‐associated marker expression.

### 2.11 Western blotting analysis

Cells and purified EVs lysis were performed as follows: whole cell lysate was lysed in RIPA buffer containing protease and phosphatase inhibitors (Sigma Aldrich Corporation), then clarified by centrifugation at 9500 ***g*** for 10 min at 4 °C, while isolated EVs were prepared in a reducing (for LGALS3BP, Alix and β‐actin immunoblotting) or non‐reducing (for CD9 and CD63 immunoblotting, without 2‐mercaptoethanol) sample buffer (50 mm Tris–HCl pH 6.8, 5% glycerol, 2% SDS, 1.5% 2‐mercaptoethanol with bromophenol blue) and heated at 95 °C for 10 min. Cellular protein amount of cellular and EVs lysates was determined using the Bradford reagent (Bio‐Rad), and equal amounts of proteins were separated on 10% or 12% SDS/PAGE and transferred to nitrocellulose membrane (Merck Millipore, Germany). The membranes were blocked in 5% skimmed milk in Tris‐buffered saline buffer with 0.1% Tween‐20 for 1 h at room temperature, followed by incubation with primary antibodies overnight at 4 °C. Subsequently, the membranes were incubated with HRP‐conjugated secondary antibodies, detected by enhanced chemiluminescence solution (Pierce, Thermo Fisher Scientific, Cambridge, MA, USA) using a chemiluminescence detection system (Bio‐Rad).

### 2.12 ELISA

For cell‐derived supernatants and EVs analysis, as previously described [31], well plates were coated with murine anti‐LGALS3BP antibody SP2 [37] (2 μg·mL^−1^) overnight at 4 °C. After blocking with 1% BSA in PBS for 1 h, 50 μL of intact EVs or PBS (as control) was added and incubated for 1 h at RT. After three washes with PBS‐0.05% Tween‐20, humanized anti‐LGALS3BP antibody 1959 (1 μg·mL^−1^) was incubated for 1 h at RT. For the detection, after three washes with PBS‐0.05% Tween‐20, anti‐human IgG‐HRP secondary antibody (A0170; Sigma Aldrich) was added (1 : 5000) and incubated for 1 h at RT. After washes, stabilized chromogen was added for at least 10 min in the dark, before stopping the reaction with the addition of 1 N H_2_SO_4_. The resulting colour was read at 450 nm with ELISA plate reader.

Serum‐derived EVs‐associated and total LGALS3BP was measured using Proteintech™ Human LGALS3BP ELISA Kit (Cod. KE00155) following the manufacturer's protocol; for serum, 1 : 10 000 dilution was used, while for serum‐derived EVs, 1 ng of total protein amount was analysed, based on Bradford dosage. Human serum‐derived EVs were quantified using ExoELISA against CD63 (Cod. EXEL‐ULTRA‐CD63‐1; SystemBiosciences, Mountain View, CA, USA) according to the manufacturer's instructions. Briefly, a standard curve was constructed using exosome standards, and test samples of purified EVs run in duplicate with exosome quantity extrapolated from the standard curve.

Also, for LGALS3BP expression analysis of mice serum‐derived EVs in correlation studies, they were analysed using Proteintech™ Human LGALS3BP ELISA Kit (Cod. KE00155, Minneapolis, MN, USA), and in this case, 100 ng of total protein amount was analysed based on Bradford dosage.

For biodistribution studies by ELISA, following sacrifice, mouse organs and tumours were immediately excised, rinsed with PBS and weighed. Each organ or tumour was placed in a 15‐mL polystyrene tube containing a volume of tissues lysis buffer (100 mm Tris–HCl pH 7.6, 150 mm NaCl, 1 mm EDTA, 1% Triton X‐100, 0.5% Sodium‐deoxycholate, phosphatase and proteinase inhibitors) in the ratio of tissue weight: buffer volume 1 : 1; and homogenized in ice with TissueRuptor® II – QIAGEN. The homogenate was centrifuged at 13 000 r.p.m. for 30 min and the supernatant was taken as the extract. Tissue uptake of intact 1959‐sss/DM4 was measured by sandwich ELISA using as capture antibody a mouse anti‐DM1/4 monoclonal antibody (1 μg·mL^−1^, Clone G2E6, Cod. CABT‐L3105; Creative Diagnostics, London, UK) and goat anti‐human IgG‐HRP conjugated secondary antibody for detection.

### 2.13 Immunohistochemistry

For the evaluation of LGALS3BP in human specimens, 5‐μm tissue sections of paraffin embedded blocks from GBM patients were stained with a goat polyclonal antibody raised against human LGALS3BP (1 : 400 dilution; 60′ incubation; R&D, cat. number #AF2226). Antigen retrieval was performed by microwave treatment at 750 W (10 min) in citrate buffer (pH 6.0). The ABC kit was used for signal amplification. 3,3′‐Diaminobenzidine was used as chromogen. Negative controls were obtained using matched isotype control antibody.

### 2.14 Statistical analysis

For ELISA data, *P* values were determined by Mann–Whitney test, for *in vivo* xenograft growth curves *P* values were determined by Mantel–Cox test and considered significant for *P < 0.05, **P < 0.005 and ***P < 0.0005. Experimental sample numbers (*n*) are indicated in the figure legends. Chi‐square test was used to investigate the relationships between LGALS3BP expression and clinicopathological parameters of patients. Youden's *J* index on receiver operating characteristic (ROC) curve was used to determine the optimal cut‐off point of LGALS3BP percentage of expression in pathological samples for discriminating between patients' surviving more than 12 months from surgery. A tissue expression of LGALS3BP of 95% was determined as the most accurate cut‐off value to differentiate between patients surviving more or less than 12 months from surgery at ROC curve (AUC 0.73; *J* 0.45). Accordingly, patients were divided into a low LGALS3BP expression group (< 95%) and high LGALS3BP expression group (≥ 95%). Kaplan–Meier survival analysis (log‐rank test) was performed to determine the possible difference in survival between the two groups.

## 3 Results

### 3.1 LGALS3BP is highly expressed in GBM

**Fig. 1:**
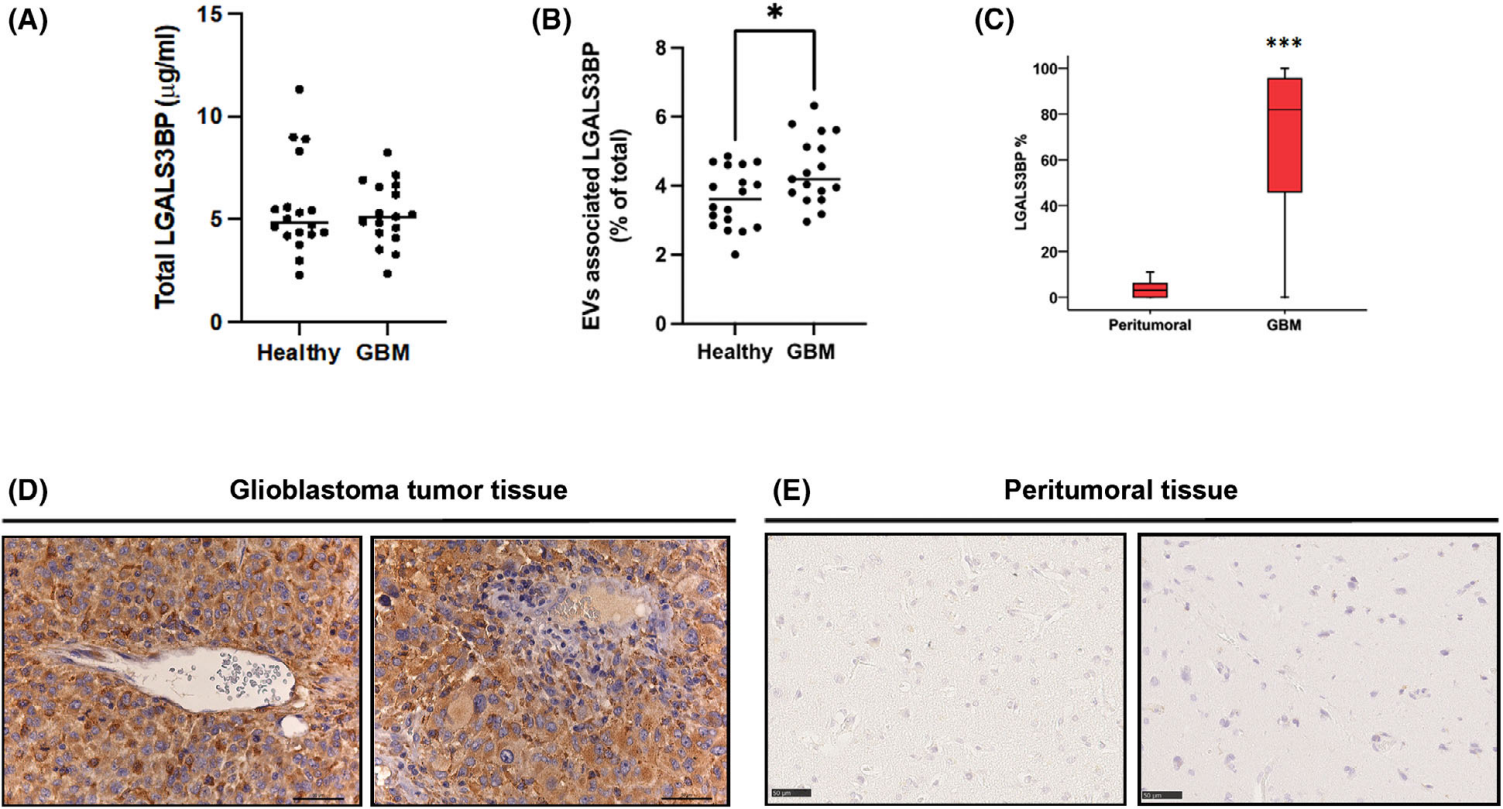
LGALS3BP expression levels in GBM patients. (A) Sandwich ELISA performed on GBM patient (*n* = 17) and healthy donor (*n* = 18) serum samples. (B) Graph showing the percentage of EVs‐associated LGALS3BP over total circulating LGALS3BP in serum. Mann–Whitney test *P = 0.03. (C) Box‐and‐whisker diagram of the distribution of LGALS3BP in GBM cases and peritumoral matched samples (*n* = 53). The upper and lower ends of boxes represent 75th and 25th percentiles. The median value is shown with a solid line. Mann–Whitney test ***P < 0.001. Data are shown as mean ± standard deviation. (D, E) Representative images of IHC staining for LGALS3BP expression in GBM and peritumoral matched samples. All IHC images have been acquired using an optical microscope at a scale bar of 50 μm.

In order to evaluate the potential role of circulating LGALS3BP as biomarker for GBM, serum‐derived EVs from healthy donors and GBM patients before surgery were isolated using a polymer‐based methodology, and purified EVs were characterized by WB and ELISA (Fig. S1A,B). Total circulating (Fig. 1A) and EVs‐associated (Fig. 1B) LGALS3BP were analysed by ELISA. Interestingly, while we observed a similar amount of total secreted LGALS3BP in the two groups, we found a significant increase (*P* = 0.03) of vesicular‐associated LGALS3BP expression in cancer samples compared to healthy donors, as calculated by the average percentage of vesicular on total circulating LGALS3BP (Fig. 1B). These results suggest an increased release of LGALS3BP‐expressing EVs by tumour cells. Furthermore, to evaluate LGALS3BP expression level in the tumour tissues, we performed the immunohistochemical (IHC) staining of 53 GBM and matched peritumoral samples (Fig. 1C). LGALS3BP was expressed, with a cytoplasmatic pattern (Fig. 1D), in 51 out of 53 (96.2%) GBM cases, with a median value of 82.5% (Fig. 1C). Of note, the protein is barely detectable in the peritumoral tissues where is primarily confined to perineuronal glial cells (Fig. 1E). However, LGALS3BP expression was not significantly correlated with any clinicopathological variables of patients (Tables S1 and S2) as well as with OS (Fig. S1C). Taken together, these observations strongly indicate that LGALS3BP is homogenously overexpressed in GBM compared to peritumoral tissues and enriched in cancer‐released EVs.

### 3.2 EVs‐associated LGALS3BP is highly expressed in patient‐derived glioblastoma cell lines

We aimed to establish preclinical models to determine the potential value of vesicular LGALS3BP as diagnostic and therapeutic target for GBM disease. Therefore, starting from fresh tumour tissues obtained after surgical resection, we derived a panel of primary GBM patient‐derived cell lines. As a first step, established cell lines were investigated for neuronal and glioma markers expression by flow cytometry analysis (Fig. S2A). Next, using doubling time as read‐out, we selected three cell lines, hereafter named Gch6, Gch10 and Gch14, which displayed a typical dendritic‐like morphology and presented numerous and extensive cytoplasmatic prolongation as filopodia (Fig. S2B,C). The three selected cell lines were then analysed for LGALS3BP content. According to IHC data on tumour tissues, we found that LGALS3BP is highly expressed at both mRNA, intracellular and secreted protein levels in all patient‐derived GBM cell lines tested (Fig. S3A–C).

**Fig. 2:**
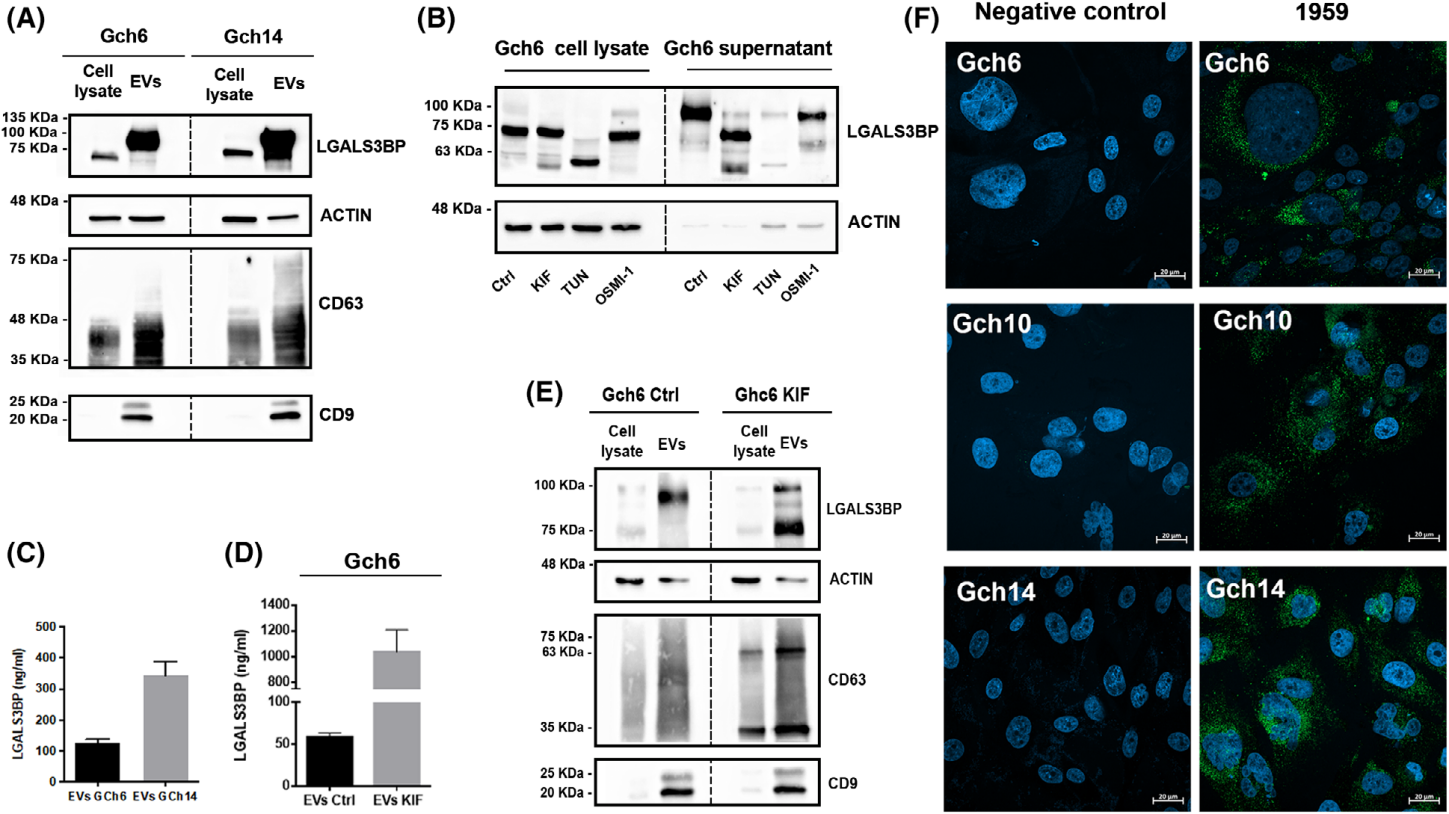
LGALS3BP expression levels in EVs isolated from GBM patient‐derived cell lines. (A) LGALS3BP and EVs markers CD9, actin and CD63 expression levels in whole lysate and EVs isolated from Gch6 and Gch14 cellular supernatants (*n* = 2). (B) Effect of N‐Glycosylation (kifunensine, KIF and tunicamycin, TUN) and O‐Glycosylation (OSMI‐1) inhibitors on the protein profile for LGALS3BP in Gch6 whole lysate and corresponding supernatant (*n* = 2). (C) Sandwich ELISA performed on intact EVs isolated from Gch6 and Gch14 cell line supernatants (*n* = 3). Data are shown as mean ± standard deviation. (D) Sandwich ELISA performed on intact EVs isolated from Gch6 treated with KIF (*n* = 3). Data are shown as mean ± standard deviation. (E) Effect of KIF treatment on the protein profile in whole lysate and EVs isolated from Gch6 (*n* = 2). (F) Confocal images of live GBM patient‐derived cells labelled with humanized 1959 anti‐LGALS3BP antibody followed by AlexaFluor 488 conjugated secondary anti‐human IgG antibody (green). Cell nuclei were stained with DAPI (blue) (*n* = 3). Images were taken at 40× magnification. Scale bar: 20 μm. Negative controls were only incubated with secondary antibody.

In order to evaluate EVs‐associated LGALS3BP expression level, we isolated EVs from supernatants of Gch6 and Gch14 cell lines, which possess faster doubling time (Fig. S2C). As shown by SDS/PAGE (Fig. 2A), we found that EVs derived from GBM cell lines resulted to be highly enriched in complete mature form of LGALS3BP with a molecular weight ranging from 90 to 100 kDa, indicating its extensive glycosylation [28, 31]. Indeed, it was reported that LGALS3BP possesses 7 N‐ and 3 O‐linked glycosylation sites [28]. Here, using different N or O‐linked glycosylation inhibitors, we confirmed that LGALS3BP undergo glycosylation modification in GBM cells (Fig. 2B). Interestingly, TUN which acts in the first step of N‐glycosylation processes reduces the molecular weight (around 60 kDa) of the intracellular form strongly inhibiting its secretion. On the contrary, treatment with KIF, an inhibitor that intervenes in spatially and temporally N‐glycosylation processes subsequent to those of tunicamycin, promotes the secretion of a non‐complete mature form of the protein (molecular weight around 75 kDa). Finally, O‐linked glycosylation inhibition through OSMI‐1 treatment resulted in the production of LGALS3BP variants differing in size possibly due to a different glycosylation profile of the secreted forms (Fig. 2B).

Using the anti‐LGALS3BP therapeutic antibody named 1959 [30], we demonstrated that intact LGALS3BP+ EVs released by GBM cells can be detected and LGALS3BP amount quantified by a sandwich ELISA assay (Fig. 2C). Interestingly, the binding of 1959 to its target antigen is not affected by the glycosylation status (Fig. 2D). Moreover, as evaluated by ELISA and confirmed by western blotting using a different primary antibody, we observed an increased amount of LGALS3BP induced by KIF treatment, (Fig. 2E), thus indicating that LGALS3BP stability may be modulated by its glycosylation status [28].

Of note, using confocal imaging, we found that the therapeutic antibody accumulates in the extracellular milieu of LGALS3BP‐expressing cells (Fig. 2F). These observations indicated that established patient‐derived GBM cells are a valuable preclinical model to study vesicular LGALS3BP as potential disease marker and therapeutic target.

### 3.3 EVs‐associated LGALS3BP correlates with tumour burden and anti‐LGALS3BP ADCs induce tumour growth inhibition in GBM patient‐derived xenograft model

**Fig. 3:**
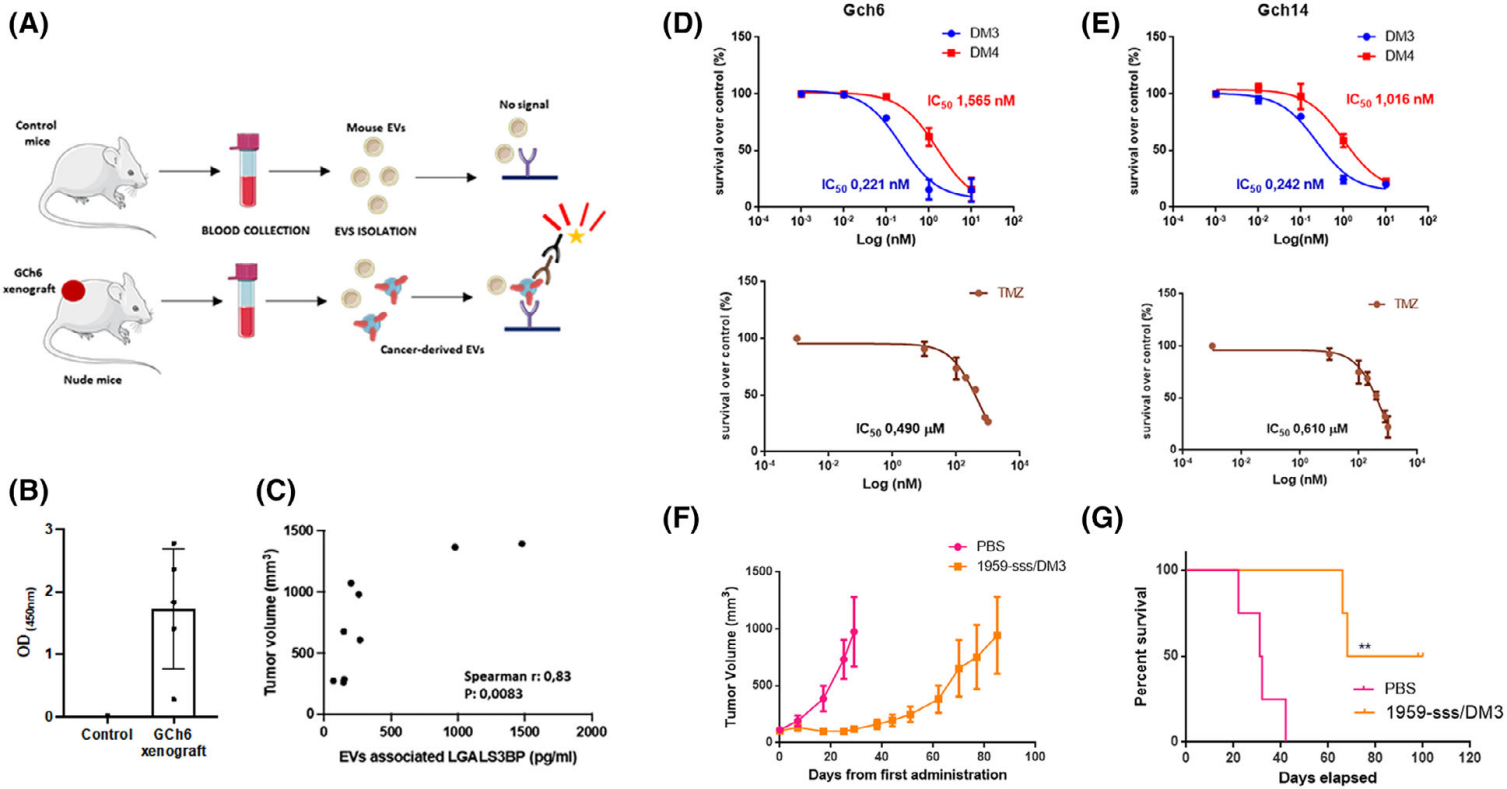
Extracellular vesicles‐associated LGALS3BP correlation with tumour volume and therapeutic activity of 1959‐sss/DM3 in GBM patient‐derived xenograft model. (A) Graphic scheme representing ELISA of human LGALS3BP on EVs in serum samples from mice with human GBM xenograft. (B) Levels of LGALS3BP on EVs isolated from the serum samples of control nude mice or mice bearing human GCh6 GBM xenograft, measured by ELISA (*n* = 5). Bar graph represents average ± standard deviation. (C) Spearman correlation between EVs‐associated LGALS3BP and tumour burden in xenograft‐bearing nude mice (*n* = 9). (D, E) Cytotoxic activity of SH‐DM3, SH‐DM4 and temozolomide (TMZ) was obtained by using the MTT assay on Gch6 and Gch14 cell lines at indicated doses after 5 days. IC50 values were determined with graphpad prism software (San Diego, CA, USA) (*n* = 3). Data are shown as mean ± standard deviation. (F) CD1 nude mice harbouring Gch6 xenografts were treated with vehicle (PBS, *n* = 4) or 1959‐sss/DM3 (10 mg·kg^−1^, *n* = 4) twice weekly as indicated by the arrows. Data are shown as mean ± standard error. (G) (PBS, *n* = 4) (10 mg·kg^−1^, *n* = 4). Kaplan–Meier survival curves. Log‐rank (Mantel–Cox) Test. **P = 0.006.

We attempted to generate subcutaneous xenograft models using established patient‐derived GCh6 and GCh14 cell lines. Unfortunately, only Gch6 cell line resulted to possess the ability to grow as subcutaneous tumour in nude mice, and therefore only this cell line model was used as *in vivo* model for further studies. As a first step to investigate the secretion of vesicular LGALS3BP by GBM cells *in vivo*, serum‐derived EVs were purified from nude mice harbouring GCh6 xenograft, characterized for EVs‐associated marker Alix by WB (Fig. S4A), and further analysed by ELISA (Fig. 3A,B). Antibodies specifically recognized human LGALS3BP on the circulating EVs isolated from mice bearing human GBM xenografts but not from vehicle (PBS) injected mice (Fig. 3B). Moreover, the level of circulating EVs‐associated LGALS3BP positively correlated with tumour burden (Fig. 3C), thus suggesting that vesicular LGALS3BP released from GBM cancer cells may be used as potential circulating disease marker.

As next step, we evaluated whether secreted LGALS3BP can be used as actionable target for ADC therapy in our preclinical model. Indeed, using the 1959 therapeutic antibody, we have previously developed a non‐internalizing anti‐LGALS3BP ADC, obtained by site‐specific conjugation of maytansine derivative tubulin inhibitors (DM3 or DM4) and designed to have a fixed DAR of 2. The ADC showed to promote tumour shrinkage in melanoma and neuroblastoma preclinical models [30, 31]. Here, we first evaluated GBM cells’ sensitivity to DM3 and DM4 drugs performing *in vitro* cytotoxic assays using temozolomide (TMZ), a clinically approved chemotherapeutic agent currently used for GBM treatment, as reference standard [6]. *In vitro*, cytotoxic assays (Fig. 3D,E) revealed that antitumour activity of both DM3 and DM4 are significantly superior (IC50 in the nanomolar range) compared to that of TMZ (IC50 in the micromolar range).

Then, we designed the first therapeutic study using 1959‐sss/DM3 [30, 31] according to previously optimized dosage and schedule, that is, twice/weekly injections of 10 mg·kg^−1^, for a total of three administrations. ADC treatments induced a significant and prolonged survival of treated mice (*P* = 0.007) although tumour re‐growth was observed after 80 days from the start of treatments (Fig. 3F,G).

**Fig. 4:**
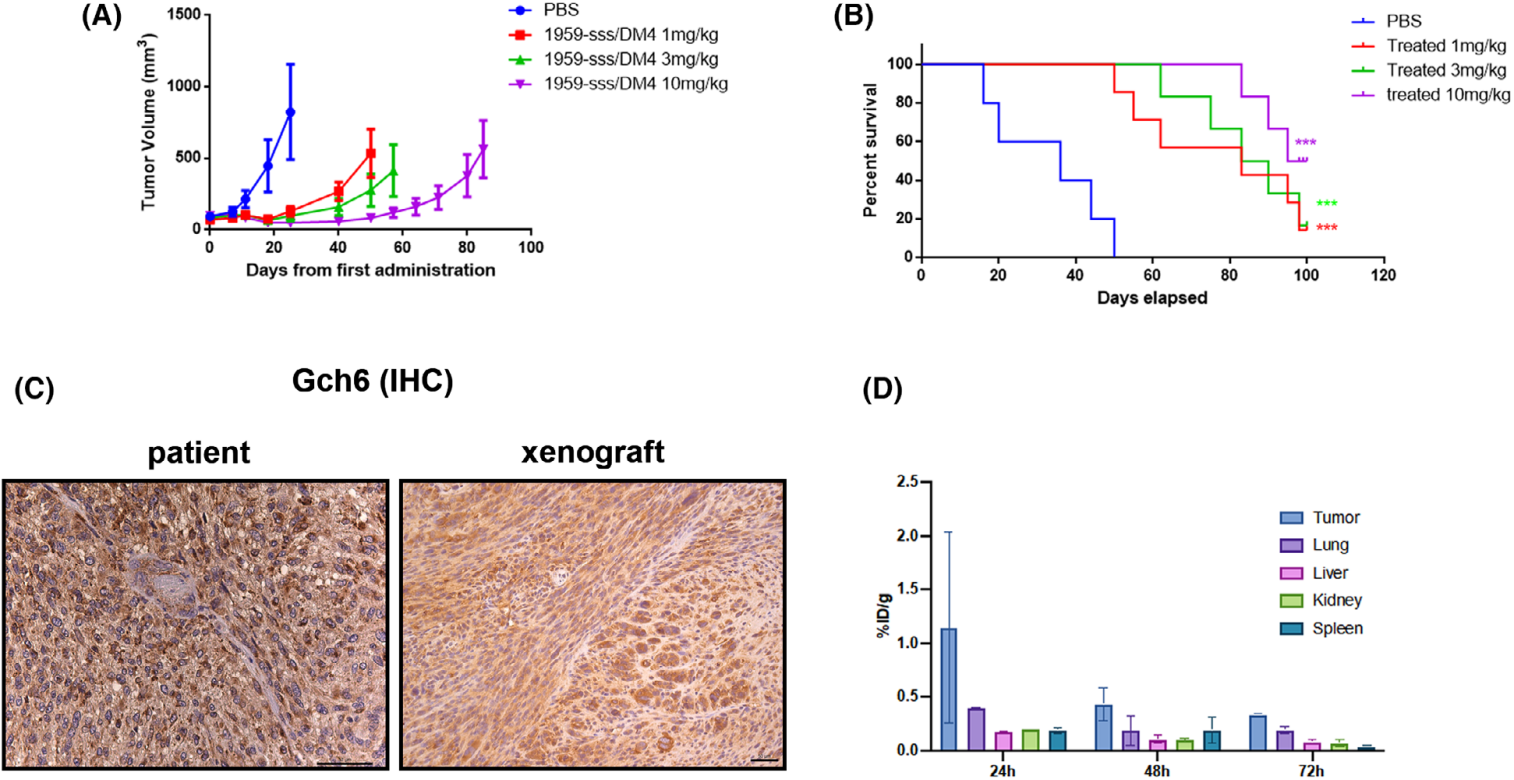
1959‐sss/DM4 dose–response efficacy and biodistribution. (A) CD1 nude mice Gch6 xenografts were treated with vehicle (PBS) or 1959‐sss/DM4 at the indicated doses twice weekly (PBS, *n* = 5; 1 mg·kg^−1^, *n* = 7; 3 mg·kg^−1^, *n* = 6; 10 mg·kg^−1^, *n* = 6). Data are shown as mean ± standard error. (B) (PBS, *n* = 5; 1 mg·kg^−1^, *n* = 7; 3 mg·kg^−1^, *n* = 6; 10 mg·kg^−1^, *n* = 6). Kaplan–Meier survival curves. Log‐rank (Mantel–Cox) Test. ***P < 0.001. (C) Representative images of IHC staining for LGALS3BP expression in Gch6 patient primary tumour and in Gch6 xenograft (*n* = 4). Scale bar: 50 μm. (D) Biodistribution of 1959‐sss/DM4 in tumour‐bearing nude mice. ADC was injected into the tail vein of nude mice bearing a xenograft of GCh6 patient‐derived cell line. After 24, 48 and 72 h, the mice were sacrificed, and the ADC uptake was determined in tissues (D) by ELISA using an anti‐DM4 antibody as coating. Each column and bar show the mean and standard deviation for three mice. Tissue uptake of ADC is expressed as percentage of injected dose per gram of tissue (%ID·g^−1^). Data are shown as mean ± standard deviation.

In particular, 1959‐sss/DM4 used at the dosage of 10 mg·kg^−1^ induced a significantly prolonged survival (*P* = 0.0003) with 50% of treated mice showing partial remission at 100 days from the start of treatment (Fig. 4B). Notably, no signs of toxicity, in terms of mice weight loss, were observed during the above described *in vivo* experiments (Fig. S4B,C), thus indicating that the ADC is well tolerated in mice. We previously demonstrated that DM3‐based ADC was stable in mice serum as no payload release was observed in treated animals [31]. Here, we attempted to quantify ADC accumulation on the tumour site. Human GBM bearing mice were treated with 10 mg·kg^−1^ of 1959‐sss/DM4 and tumour and normal tissues were collected at 24, 48 and 72 h after ADC administration. Samples were then analysed by ELISA using an anti‐DM4 antibody as capture antibody (Fig. 4D). ELISA analysis showed that intact ADC preferentially accumulates at the tumour site compared to other normal tissues, such as lung, liver, kidney and spleen (Fig. 4D). Maximum accumulation of the intact ADC was observed at 24 h followed by a significant decrease over the other time‐points (48 and 72 h), thus suggesting that the ADC is catabolized on the tumour site inducing payload release from the antibody backbone.

## 4 Discussion

Glioblastoma multiforme, the most common and aggressive primary brain cancer in adults, is a deadly tumour where the hindrance to have an early diagnosis and the lack of an effective treatment makes the disease incurable [2, 7]. Despite the significant clinical progress obtained for other aggressive indications, including lung, breast and colorectal cancer, GBM average OS still remains very poor, ranging from 9 to 12 months [11, 12]. Indeed, the majority of GBM patients are diagnosed at late stage and nearly all of them experience relapse after surgery followed by chemoradiation therapy [2, 10]. Hence, increasing the chances of getting an early diagnosis, as well as expanding the available therapeutic arsenal is an urgent medical need for GBM patients.

LGALS3BP is a glycosylated protein overexpressed in a variety of human malignancies [28] including GBM [38], being one of the most abundant surface components of cancer‐derived EVs [39, 40, 41, 42, 43, 44, 45, 46, 47]. High expression of vesicular LGALS3BP was found to be associated with poor prognosis in ovarian cancer [39]. Moreover, vesicular LGALS3BP expression levels measured in patient plasma correlate with glioma grade and progression [29]. In our work, we have evaluated LGALS3BP expression in a cohort of 53 GBM patients by IHC. Although LGALS3BP expression was not correlated with any clinicopathological variables and OS in our series of patients, we observed a massive and homogenous expression in 96% of patient tissues while peritumoral regions resulted to be nearly negative (Fig. 1D,E). Moreover, we found that the amount of EVs‐associated LGALS3BP increases in patients compared to healthy donors (Fig. 1B). Importantly, we demonstrated that LGALS3BP level detected in EVs isolated in serum of mice harbouring human GBM xenograft significantly correlated with tumour burden (Fig. 3C). Our findings reinforce observation recently achieved in the context of the use of vesicular markers for liquid biopsy [17, 18]. In addition, it has been recently shown that protein expression of EGFR in serum EVs is an effective diagnostic marker of glioma [48]. Indeed, the role of vesicular components appears particularly relevant in the context of the GBM, where a series of seminal studies have revealed the importance of cancer released EVs in tumour biology and acquisition of resistance [14, 49, 50, 51]. Although our results show a significant increase of vesicular‐associated LGALS3BP expression in GBM patients as compared to healthy controls, further studies in a larger series of patients are required to validate EVs‐associated LGALS3BP as a potential circulating marker for GBM. Moreover, a characterization of LGALS3BP glycosylation pattern could be useful to discriminate whether the vesicular forms isolated from cancer patients may differ from that obtained from healthy samples, thus increasing the specificity of LGALS3BP as a GBM associated marker.

A second important aspect our work suggests that extracellular LGALS3BP can be efficiently targeted by a specific non‐internalizing antibody–drug conjugate. Indeed, we have recently reported that high expressing LGALS3BP melanoma and neuroblastoma can be effectively treated with DM3 and DM4 maytansinoid‐based ADCs directed against LGALS3BP [30, 31]. Importantly, our previous data indicate that only the combination of the payload with the 1959 antibody is effective, as neither the cytotoxic agent alone nor the naked antibody showed therapeutic activity [31].

Here, we extended our previous studies showing that 1959‐sss/DM4 ADC potently inhibits *in vivo* the growth of a GBM patient‐derived cell line and significantly increases the survival of treated mice in a dose‐dependent manner (Fig. 4A,B). The ADC showed a favourable tumour accumulation with no sign of toxicity (Fig. 4D; Fig. S4B,C). In principle, the lack of adverse effects observed in our mouse preclinical model could be not predictive of potential off‐target toxicity of the ADC. However, additional evidence about 1959sss‐based ADC tolerability has been obtained with an acute toxicity study on rabbits [30]. This latter study is of particular relevance in terms of 1959‐based ADCs safety, as the rabbit the only species, among 15 examined where the antibody completely cross‐reacts with man.

It is important to highlight that the high therapeutic activity obtained with our ADC using DM4 as payload is particularly relevant as this drug is being used in the design of several ADCs currently under clinical trial investigations, and therefore this will eventually facilitate its further clinical development.

A limitation of our findings may be represented by the fact that subcutaneous model was used for the therapeutic study. Indeed, the principal obstacle for therapies against GBM resulted to be the BBB, a highly specialized non‐fenestrated physical barrier formed by microvascular endothelial cells interconnected by multi‐protein tight junctions consisting of claudins, occludins and junctional adhesion molecules [52]. However, a growing bulk of studies have provided evidence that the BBB – both at baseline and in the context of malignancy – is permissive for mAbs, thus providing a rationale for their use in treating intracranial malignancy [53, 54, 55].

## 5 Conclusions

Results obtained in the present study strongly encourage to validate the therapeutic activity of the ADC in more valuable and physiological setting such as orthotopic xenografts. Moreover, the development of such models would be diriment to establish the full potential of vesicular LGALS3BP as potential disease marker, therapeutic target and circulating biomarker for therapy response. Overall, our data pave the way for further preclinical and clinical studies to assess the role of vesicular LGALS3BP as potential biomarker and therapeutic target in GBM.

## Conflict of interest

Stefano Iacobelli and Gianluca Sala are shareholders of Mediapharma srl.

## Author contributions

BD performed the experiments, interpreted data and read and corrected the paper; EC performed the experiments, interpreted data and read and corrected the paper; SP performed purification of the antibody, conjugation and analytic characterization of the ADC; RL performed IHC work; PL performed cytofluorimetry experiments; VDL was involved in funding acquisition, critically interpreted data and revised the manuscript for important intellectual content; FG supervised ADC generation, read the paper and suggested ideas; SI was involved in conceptualization, supervision, and writing‐editing manuscript; RI supervised ADC generation and critically revised the manuscript for important intellectual content; AM collected patient sample, suggested ideas and read and corrected the paper; GT collected patient sample, suggested ideas and wrote the paper; GS designed the study, supervised the project and wrote the paper.

### Peer Review

The peer review history for this article is available at https://www.webofscience.com/api/gateway/wos/peer‐review/10.1002/1878‐0261.13453.

## Supporting information


**Fig. S1.** Characterization of serum‐derived EVs and overall survival in GBM.

**Fig. S2.** Characterization of the GBM patience‐derived cell lines.

**Fig. S3.** GBM patient‐derived cell lines characterization.

**Fig. S4.** Characterization of serum‐derived EVs and mice body weight.

**Table S1.** Clinicopathological characteristics of GBM patients.
**Table S2.** LGALS3BP status according to the clinicopathological features of glioblastoma patients.

## Data Availability

All data generated or analysed during this study are included in this published article.
