# Supplementary figures and images for: Extracellular LGALS3BP: a potential disease marker and actionable target for antibody–drug conjugate therapy in glioblastoma
Source: Mol Oncol. 2023 Jun 7;17(8):1460–73. doi: 10.1002/1878-0261.13453 (PMC10399712; doi:10.1002/1878-0261.13453)

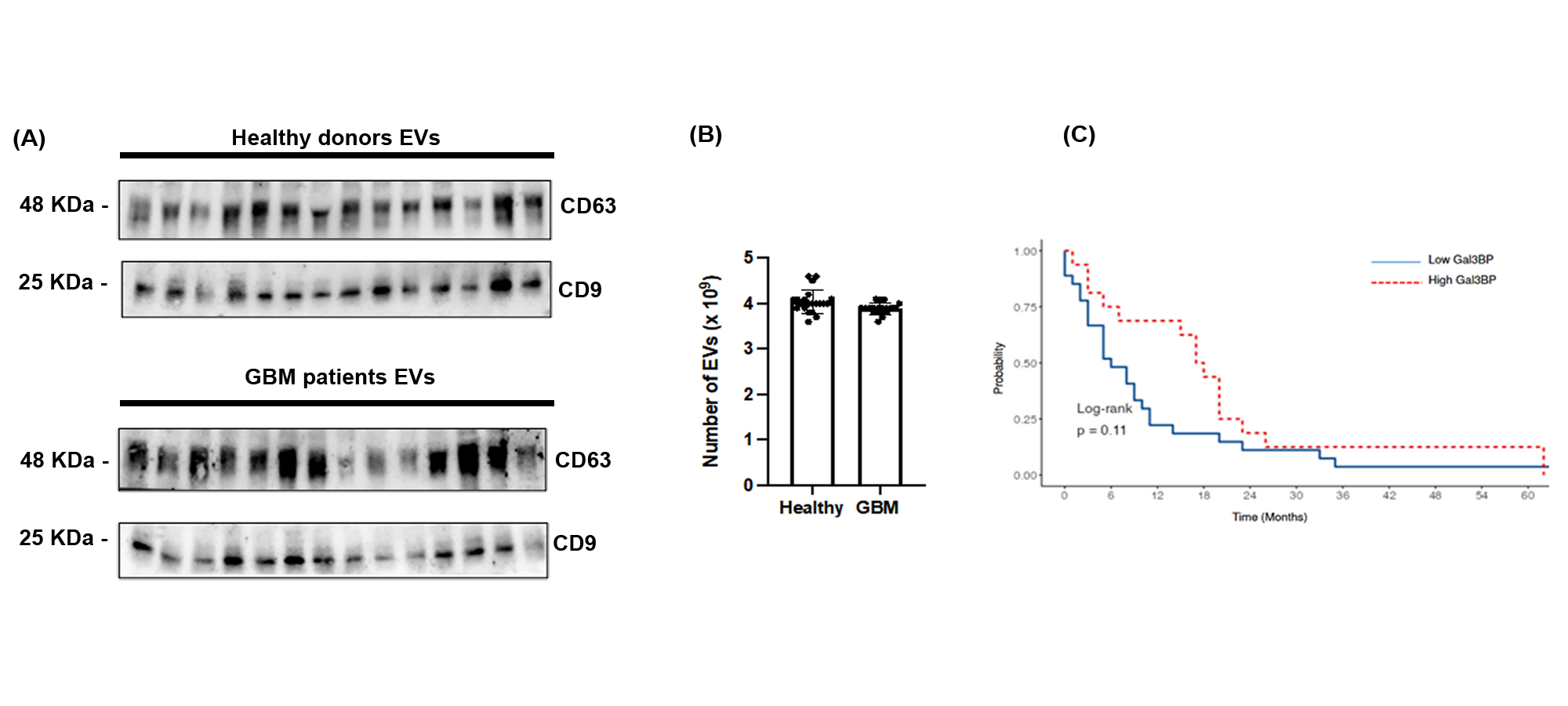

Supplement: Supplementary file 1 — Fig. S1. Characterization of serum‐derived EVs and overall survival in GBM. [file MOL2-17-1460-s001.tif]

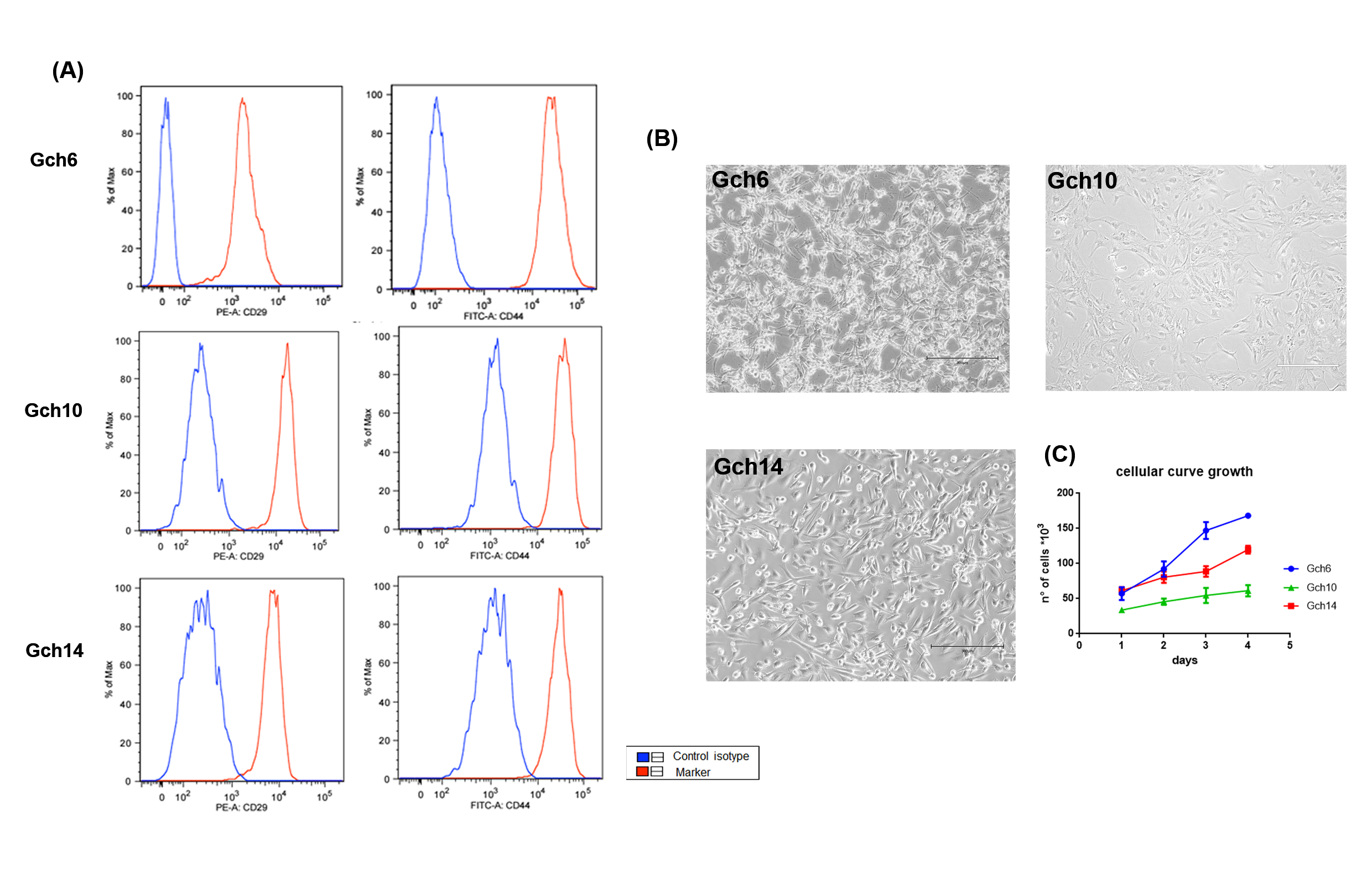

Supplement: Supplementary file 2 — Fig. S2. Characterization of the GBM patience‐derived cell lines. [file MOL2-17-1460-s005.tif]

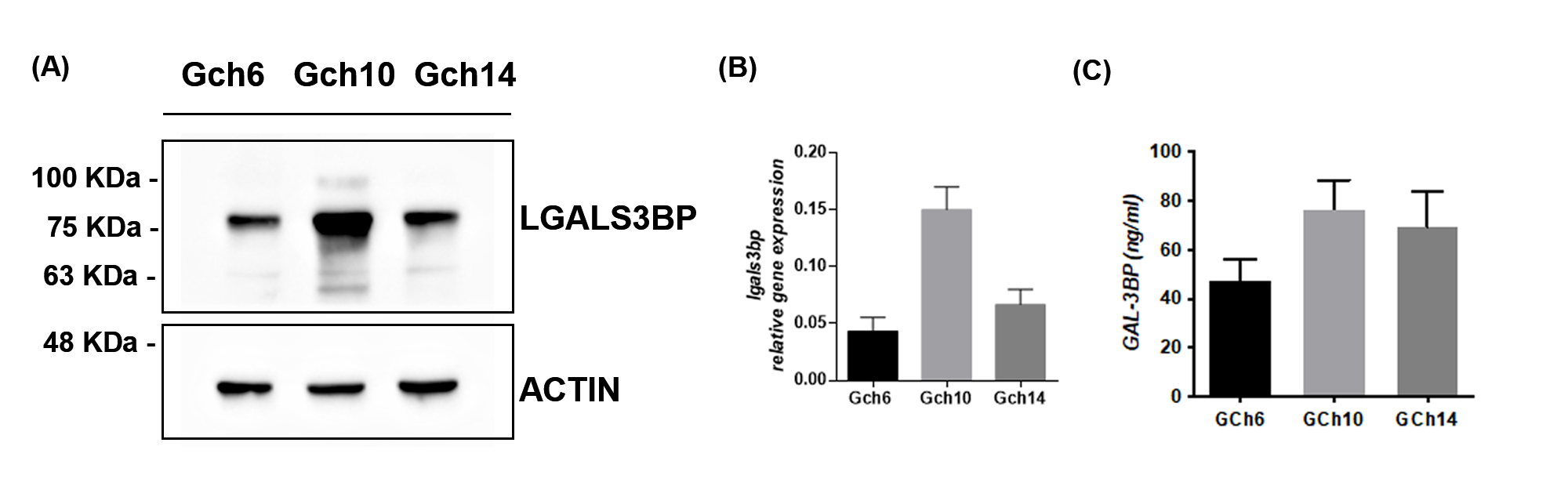

Supplement: Supplementary file 3 — Fig. S3. GBM patient‐derived cell lines characterization. [file MOL2-17-1460-s003.tif]

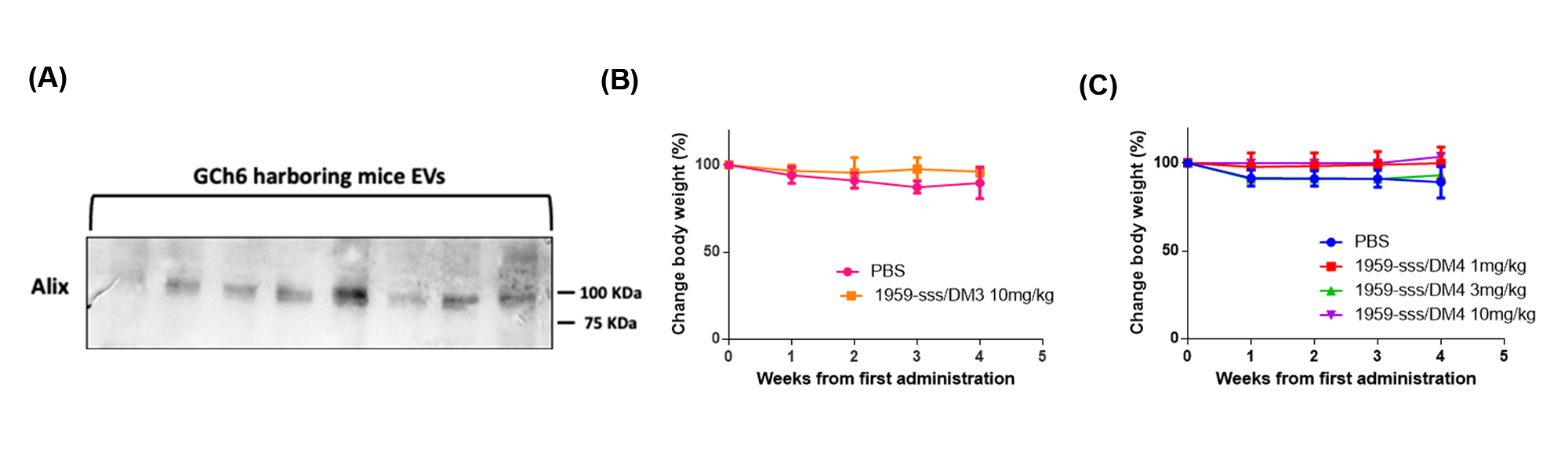

Supplement: Supplementary file 4 — Fig. S4. Characterization of serum‐derived EVs and mice body weight. [file MOL2-17-1460-s002.tif]
