# Supplementary material for: Extracellular LGALS3BP: a potential disease marker and actionable target for antibody–drug conjugate therapy in glioblastoma
Source: Mol Oncol. 2023 Jun 7;17(8):1460–73. doi: 10.1002/1878-0261.13453 (PMC10399712; doi:10.1002/1878-0261.13453)
Supplement: Supplementary file 5 — Table S1. Clinicopathological characteristics of GBM patients. Table S2. LGALS3BP status according to the clinicopathological features of glioblastoma patients. [file MOL2-17-1460-s004.docx]

**Supplementary Figure Legends**

**Supplementary Figure 1: Characterization of serum-derived extracellular vesicles** **(EVs) and overall survival in GBM** (A) Immunoblot showing EVs-associated markers CD63 and CD9 expression in EVs isolated from GBM patients and healthy donors’ sera, (*n*=2). (B) Quantification of EVs isolated from serum performed by CD63 ExoELISA. Data are shown as mean ± standard deviation. (C) Kaplan-Meier curves showing no significant difference in survival from surgery between patients showing a high or low expression of tissue LGALS3BP at pathological analysis.

**Supplementary Figure 2: Characterization of the GBM patience-derived cell lines.** (A) Representative histograms of the flow cytometry analysis of GBM-associated protein expression in established Gch6, 10 and 14 GBM patience-derived cell lines. (B) Representative images of Gch6, Gch10 and Gch14 cell lines. Scale bar: 300 µm. (C) cellular curves growth of Gch6, Gch10 and Gch14, doubling times were calculated using formula Δt × [ln2/(lnNt − lnN0)], where Δt is the duration of cell proliferation (exponential phase) in hours, and N0 and Nt are the respective numbers of cells at the beginning and end of this period; Gch6 doubling time 33 hours, Gch10 doubling time 81 hours and Gch14 doubling time 75 hours, (*n*=3).

**Supplementary Figure 3: GBM patient-derived cell lines characterization.** (A-C) Gene, intracellular and secreted LGALS3BP protein levels analyzed by RT-PCR, WB, ELISA in Gch6, 10 and 14 cell lines, (*n*=2). Data are shown as mean ± standard deviation.

**Supplementary Figure 4:** **Characterization of serum-derived extracellular vesicles (EVs) and mice body weight.**

(A) Immunoblot showing EVs-associated marker Alix expression in EVs isolated from sera of mice harboring GCh6 xenograft, (n=2). (B,C) Body weight of mice during the four weeks after start of treatments with 1959-sss/ADCs. Data are shown as mean ± standard error.

**Table 1.** Clinicopathological characteristics of GBM patients (n = 53)

| **Variable** | **Value (%)** |
| --- | --- |
|  |  |
| Age at diagnosis (yr) |  |
| Mean ± SD | 63.8 ± 12.1 |
| Median Range | 66.0 (25-84) |
| > 40 | 50 (94.3) |
| < 40 | 3 (5.7) |
|  |  |
| Gender |  |
| Male | 36 (67.9) |
| Female | 17 (32.1) |
|  |  |
| KPS before surgery |  |
| > 80 | 15 (28.3) |
| 50-80 | 35 (66.0) |
| < 50 | 2 (3.8) |
| Unknown | 1 (1.9) |
|  |  |
| KPS after surgery |  |
| > 80 | 16 (30.2) |
| 50-80 | 32 (60.4) |
| < 50 | 4 (7.5) |
| Unknown | 1 (1.9) |
|  |  |
| Tumor Location (Emisphere) |  |
| Left | 29 (54.7) |
| Right | 24 (45.3) |
|  |  |
| IDH |  |
| Wild-type | 51 (96.2) |
| Mutated | 2 (3.8) |
|  |  |
| MGMT |  |
| Unmetilated | 22 (41.5) |
| Metilated | 24 (45.3) |
| Unknown | 7 (13.2) |
|  |  |
| Adjuvant Therapy |  |
| No | 9 (17.0) |
| Temozolomide plus Radiotherapy | 30 (56.6) |
| Temozolomide | 4 (7.5) |
| Radiotherapy | 5 (9.4) |
| Chemiotherapy | 5 (9.4) |
|  |  |

**Table 2.** LGALS3BP status according to the clinicopathological features of glioblastoma patients.

|  |  |  |  |
| --- | --- | --- | --- |
| **Variable** | **Gal3BP** | | **p** |
|  | **Low: n (%)** | **High: n (%)** |  |
|  |  |  |  |
|  |  |  |  |
| Gender |  |  |  |
| Male | 17 (63.0) | 19 (73.1) | 0.559 |
| Female | 10 (37.0) | 7 (26.9) |  |
|  |  |  |  |
| KPS before surgery |  |  |  |
| > 80 | 9 (34.6) | 6 (23.1) | 0.651 |
| 50-80 | 16 (61.5) | 19 (73.1) |  |
| < 50 | 1 (3.8) | 1 (3.8) |  |
|  |  |  |  |
| KPS after surgery |  |  |  |
| > 80 | 8 (30.8) | 8 (30.8) |  |
| 50-80 | 14 (53.8) | 18 (69.2) |  |
| < 50 | 4 (15.4) | 0 (0.0) | 0.105 |
|  |  |  |  |
| Tumor Location (Emisphere) |  |  |  |
| Left | 13 (48.1) | 16 (61.5) |  |
| Right | 14 (51.9) | 10 (38.5) | 0.412 |
|  |  |  |  |
| IDH |  |  |  |
| Wild-type | 25 (92.6) | 26 (100.0) |  |
| Mutated | 2 (7.4) | 0 (0.0) | 0.491 |
|  |  |  |  |
| MGMT |  |  |  |
| Unmetilated | 12 (48.0) | 10 (47.6) |  |
| Metilated | 13 (52.0) | 11 (52.4) | 1.000 |
|  |  |  |  |
| Adjuvant Therapy |  |  |  |
| No | 6 (24.0) | 3 (13.0) |  |
| Temozolomide plus Radiotherapy | 12 (48.0) | 18 (78.3) |  |
| Temozolomide | 4 (16.0) | 0 (0.0) |  |
| Radiotherapy | 3 (12.0) | 2 (8.7) | 0.097 |
|  |  |  |  |
